# Offshore Earthquakes Do Not Influence Marine Mammal Stranding Risk on the Washington and Oregon Coasts

**DOI:** 10.3390/ani8020018

**Published:** 2018-01-26

**Authors:** Rachel A. Grant, Anna Savirina, Will Hoppitt

**Affiliations:** 1 Department of Life Sciences, Anglia Ruskin University, East Rd, Cambridge CB1 1PT, UK; anna.savirina@hotmail.co.uk; 2 School of Biology, University of Leeds, Leeds LS2 9JT, UK; w.j.e.hoppitt@leeds.ac.uk

**Keywords:** marine mammal, mass stranding, stranding, cetacean, earthquakes, seismic activity

## Abstract

**Simple Summary:**

Marine mammals stranding on coastal beaches is not unusual. However, there appears to be no single cause for this, with several causes being probable, such as starvation, contact with humans (for example boat strike or entanglement with fishing gear), disease, and parasitism. We evaluated marine mammal stranding off the Washington and Oregon coasts and looked at offshore earthquakes as a possible contributing factor. Our analysis showed that offshore earthquakes did not make marine mammals more likely to strand. We also analysed a subset of data from the north of Washington State and found that non-adult animals made up a large proportion of stranded animals, and for dead animals the commonest cause of death was disease, traumatic injury, or starvation.

**Abstract:**

The causes of marine mammals stranding on coastal beaches are not well understood, but may relate to topography, currents, wind, water temperature, disease, toxic algal blooms, and anthropogenic activity. Offshore earthquakes are a source of intense sound and disturbance and could be a contributing factor to stranding probability. We tested the hypothesis that the probability of marine mammal stranding events on the coasts of Washington and Oregon, USA is increased by the occurrence of offshore earthquakes in the nearby Cascadia subduction zone. The analysis carried out here indicated that earthquakes are at most, a very minor predictor of either single, or large (six or more animals) stranding events, at least for the study period and location. We also tested whether earthquakes inhibit stranding and again, there was no link. Although we did not find a substantial association of earthquakes with strandings in this study, it is likely that there are many factors influencing stranding of marine mammals and a single cause is unlikely to be responsible. Analysis of a subset of data for which detailed descriptions were available showed that most live stranded animals were pups, calves, or juveniles, and in the case of dead stranded mammals, the commonest cause of death was trauma, disease, and emaciation.

## 1. Introduction

The causes of marine mammal stranding events are complex, often multi-factorial, and generally not well understood. Odell [1] warns that it is important not to generalise about the causes of stranding, as it is unlikely that there is a single causal factor that can be applied to all stranding events. However, some probable causes have been identified. Where there was a known cause, half of all marine mammal stranding events off the coast of California from 1991 to 2008 were attributable to toxins that were produced by algal blooms of the planktonic species *Pseudo-Nitzschia* spp. accumulating through the food chain [2,3,4]. Disease, parasitism, and starvation are also a major cause of dead-stranded marine mammals [5].

Brownell et al. [6], however, ruled out toxic algal blooms, as well as other factors such as moon phases, magnetic field shifts, disease, trauma, fisheries causes, and starvation in mass stranding events of pygmy killer whales, *Feresa attenuata* off the coast of Taiwan, over a 38 year period, and suggested anthropogenic sound as a probable cause. Many other authors have attributed anthropogenic sound, in particular naval activity, as a possible cause of mammal stranding events [7]. Other anthropogenic causes include gunshot, boat strikes, or entanglement [8]. The exact location that marine mammals strand may be some distance from the original source of disturbance and/or mortality event and may depend on secondary factors, such as ocean currents, coastal topography, and wind forcing.

Offshore earthquakes are a source of intense sound and disturbance [7], and therefore may be a contributing factor to stranding probability. The seismic energy released by offshore earthquakes is sharp in onset (explosion-like), can last from seconds to minutes and is coupled into tertiary (T) phase acoustic waves, which can have a frequency in excess of 100 Hz and can result in noise of up to 40 Db above background [9]. While sounds produced from earthquakes can propagate long distances through the ocean, in shallow waters much of the acoustic energy is absorbed by the sea bed, resulting in attenuation as sound waves approach coastal shelves [10]. This weak propagation of sound from the epicentral regions upwards towards the coastline is likely to mean that acoustic waves are greatly reduced in both amplitude and frequency by the time that they reach the coast [10], so this may not a very plausible mechanism for any effect of earthquakes on stranding. The website “deafwhale” [11] puts forward a hypothesis that is related to disturbance from offshore earthquakes damaging the navigational system of cetaceans through barotrauma and causing them to strand, but the hypothesis has not been empirically tested and firm evidence for this effect is lacking.

Another hypothesis put forward [12] in the case of a mass stranding of pilot whales is that magnetic field shifts caused by undersea earthquakes disorient the animals, which rely on magnetic information to navigate, causing them to strand. This is more plausible as earthquake preparation zones generate electromagnetic emissions over a wide frequency range, which presumably affect animals [13]. Furthermore, gases such as carbon monoxide and carbon dioxide can be released in large amounts during earthquakes and could kill or injure animals [13]. Of course, animals disorientated by a large earthquake may be forced into random directionless movement in any direction but where this movement coincides with a stretch of coastline, stranding may be the result. Alternatively, animals killed or injured by an earthquake may strand dead on nearby coastlines due to ocean currents and topography. It is unknown whether animals in close proximity to an offshore earthquake would strand alive or dead, but the hypotheses so far proposed (barotrauma and magnetic disorientation) would probably cause live stranding within a day or two of the seismic event, whilst gasses and asphyxiation could cause dead stranding.

Pinnipeds and cetaceans may be affected differently by earthquakes as the former are partly terrestrial. An anecdotal report of California Sea Lions leaving the San Francisco Bay area and moving up the coats to Washington and Oregon (an event unprecedented in decades) and returning only after a large offshore earthquake in the region (*pers obs*) leads to the question of whether pinnipeds are somehow affected by preseismic cues which may make them more, or less likely to strand. Kirschvink [14] puts forward the hypothesis that marine mammals generally may be less likely to strand due to offshore earthquakes as they may sense environmental cues and leave coastal areas. There are numerous pre-seismic cues that animals could potentially respond to in an earthquake preparation zone, such as positive ions, magnetic disturbances and gasses such as CO released from the fault [13], all of which have been shown to occur prior to earthquakes and whose effects on marine mammals are currently unknown [13]. In general, the rationale behind marine mammal stranding being caused by earthquakes is relatively weak, nevertheless it is important to test the hypothesis to rule out unlikely or overly speculative accounts of the causes of marine mammal stranding events.

Although there has been much speculation on this topic, little has been written in the peer reviewed literature. Raghunathan et al. [12] describe a magnitude 4.7 earthquake occurring at 13.783° N, 96.225° E off the coast of Myanmar on 21st October 2012 and a mass stranding of pilot whales the same day at Elizabeth Bay, North Andaman coast, 359 km away from the epicentre. However, Wright et al. [15] ruled out earthquakes as a cause of a mass stranding of harbour porpoise in Danish waters in 2005 as the only seismic events in the previous three weeks were below a magnitude of 2. Recently, a report on the responses of Atlantic bottlenose dolphins at the National Aquarium during the 2011 Virginia earthquake [16] showed that dolphins were able to perceive P-waves 22 s before the earthquake, and changed their behaviour to rapid swimming in echelon formation.

Many reports on the causes of marine mammal stranding are based on small sample sizes or single observations, are anecdotal or pseudoscientific accounts, or contain speculation as to causal factors. Media reports [17,18] also fuel speculation on a causal link between marine mammal stranding events and earthquakes. Bradshaw et al. [19] make the case for a reduction in speculation on the cause of marine mammal stranding and an increase in empirical hypothesis-testing approaches. To our knowledge the hypothesis that offshore earthquakes affect stranding probability has not previously been tested. In this paper, we test the premise that earthquakes occurring in the Cascadia subduction zone off the west coast of the United States affect the probability of both single and large marine mammal stranding events along that coast using large data sets collected over a six year period. In this paper, we do not attempt to explain particular mass stranding events of cetaceans, instead we test the hypothesis that offshore seismicity makes an overall difference in the probability of stranding risk of marine mammals generally.

## 2. Materials and Methods

**Figure 1:**
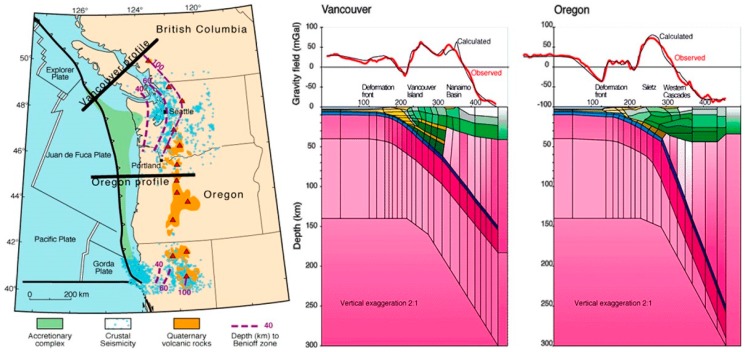
**Left**: The Cascadia subduction zone; **Right**: Oregon and Vancouver profiles. Source: (United States Geological Survey).

**Figure 2:**
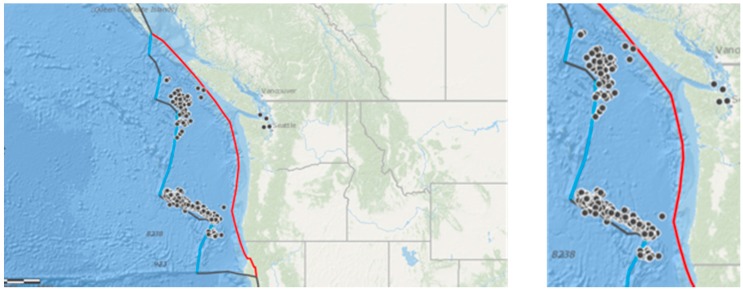
**Left**: The Cascadia basin showing subduction (red line) and a collection of transform faults (black lines) superimposed with earthquakes M > 4 (points) between 2005 and 2010; **Right**: the study area. Maps produced by ARCGIS online (Copyright © 1995–2017 Esri).

The area off the coast of Washington and Oregon is characterized by the Cascadia Subduction zone, a “megathrust” slip dip fault, which stretches for approximately 1000 km from Vancouver Island to northern California [20]. The Juan de Fuca oceanic plate is subducting beneath the North American Plate (Figure 1 and Figure 2), giving rise to frequent offshore earthquakes [21]. These earthquakes are mainly generated by the Blanco transform fault zone, a right lateral transform fault, running northeast off the coast of Oregon where the subducting Juan de Fuca plate pulls away from the Pacific plate, as well as a series of transform faults further to the north where the Explorer plate pulls away from the Pacific plate (Figure 1 and Figure 2). The location of these active faults near to a long stretch of coastline where marine stranding events are routinely recorded by the US Government (Figure 2), and the availability of six years’ robust, comprehensive datasets for the region makes it useful for testing the current hypothesis.

### 2.1. Marine Mammal Stranding Data

Marine mammal stranding data were supplied courtesy of the National Oceanic and Atmospheric Administration’s (NOAA) Marine Mammal Health and Stranding Response Program database, which is housed online at: https://mmhsrp.nmfs.noaa.gov/mmhsrp/. Records of all available marine mammal stranding events recorded off the coasts of Washington and Oregon were obtained, including those occurring in inland waters. We defined a large stranding as more than five animals beaching on the same day. This is different from the commonly accepted definition of a mass stranding that is two or more animals of the same species at the same location (NOAA). Earthquake preparation zones can be hundreds to thousands of km, depending on magnitude [22] and marine mammals can strand very far from their origin. In the case of detecting an association with earthquakes, we felt the date of stranding rather than the precise location to be important. It is entirely possible that an offshore earthquake could cause multiple animals of various species to strand at different locations. We therefore categorised large strandings as more than five animals stranding on the same day.

The available data for the region comprised records of stranding from 1999 and 2010. The number of stranding events and large stranding events recorded increased dramatically over the period of the survey (Supplementary Materials Figure S1) with no large stranding events being observed from 1999–2004. This was almost certainly due to an increase in sampling effort over the period of the study. Consequently, we only used data from 1st January 2005 to 31st December 2010 in our analysis, and included ‘year’ as a predictor in the statistical model (see below) to allow for differences in the sampling effort between years.

**Figure 3:**
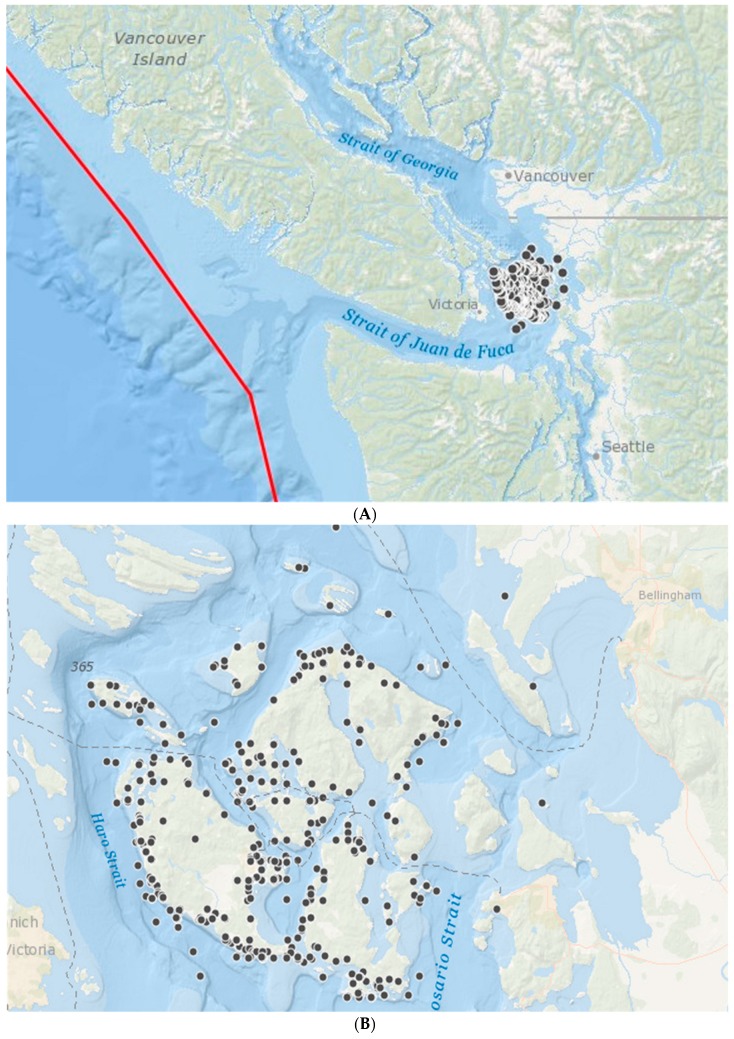
The black dots show the location of marine mammal stranding or mortality events collected by the San Juan County Marine Mammal Stranding Network between 2005 and 2010. (**A**) showing the location of the convergent plate boundary (red line); (**B**) showing detail of the San Juan Islands. Map produced in ARCGIS online (Copyright © 1995–2017 Esri).

To further elucidate whether the causes of stranding events could be caused by offshore earthquakes in the vicinity of the Cascadia Subduction Zone, we examined a subset of data from the north of Washington State, where 604 records of marine stranding were collected over five years, from April 2005 to April 2010. These data were collected by the San Juan County Marine Mammal Stranding Network (run by The Whale Museum) and cover the San Juan Islands between the Straight of Georgia and the Strait of Juan de Fuca in close proximity to the plate boundary (Figure 3).This dataset had information on cause of death and whether stranding was alive or dead, which was not available in our main dataset.

### 2.2. Earthquake Data

There were frequent earthquakes arising in the Cascadia basin over the study period. Minor earthquakes of magnitude (M) < 4 are, although frequent, so small as to be generally considered negligible and rarely affect animals [23]. Earthquake magnitude scales are logarithmic with a ten-fold increase in magnitude per scale point. Most studies considering animals in relation to seismic activity use a lower threshold of magnitude of 4 or 4.5. Including all occurring earthquakes would not be possible due to the enormous frequency of very small and inconsequential events in a seismic risk zone such as Cascadia. Therefore, only earthquakes of M > 4 were included in this analysis. The United States Geological Survey records every earthquake occurring globally, and earthquake records were collected from USGS Earthquake Archive Search and URL Builder (http://earthquake.usgs.gov/earthquakes/search/) for the Northwest Region (50.0000, 42.0000, to −130.0000, −122.0000) between 2005 and 2010 of M > 4 (Figure 2). The search area extended to more than 1000 km off the coasts of Washington and Oregon, and is indicated on the right of Figure 2. In total, there were 161 earthquakes of M > 4 during the study period (2005–2010 inclusive).

### 2.3. Data Analysis

We formulated four custom statistical models to assess whether earthquakes influence stranding probability.

The first model (a) was intended to assess whether one or more large stranding events (as defined above) were more likely to occur on the day of an earthquake, and/or on a given day in the periods of seismic activity preceding and following an earthquake. We also fitted two additional models similar to that described above, with different dependent variables: (b) whether at least one stranding was reported on each day (binary); and, (c) the number of mammals reported stranded on each day (count). It is conceivable that earthquakes might have an inhibitory effect on mass stranding events if, for example, marine mammals respond to seismic activity by moving away from land or the site of the earthquake [14]. We therefore fitted an alternative version of the model (d) with an inhibitory effect (see Supplementary Materials and Figure S3). Here, we provide an outline of the models and full model specifications and results are given in the Supplementary Material. We also carried out an analysis for cetaceans separately to account for the possible differences in stranding effects due to physiology.

The null model (no effect of earthquakes on large stranding events) was a standard logistic regression, modelling the probability that at least one large stranding would occur on any given day. Year was included as a factor to account for the differences in survey effort between years. This rules out the possibility that any apparent effect of earthquakes is a result of a chance correlation between survey effort and the number of earthquakes in a year. Season (autumn, winter, spring, or summer) was also included as a factor to account for seasonality in both mass stranding events and survey effort. Finally we allowed for the probability that one or more large stranding events occurred on a given day to depend on whether there had been any large stranding events on the previous day, to account for temporal autocorrelation.

The null model also provided the baseline rate of large stranding events in the full model, i.e., the probability of a large stranding on a given day that was not caused by an earthquake. The full model assumed that, independent of the baseline large stranding events, there were large stranding events caused by earthquakes or associated seismic activity. The model included a parameter, E, which determined the odds (on a log scale) that an earthquake would cause at least one large stranding on the day that the earthquake occurred. Additionally, since it is not known a priori how long such an effect might extend before or after an earthquake, the model assumed that the effect built up exponentially in the period preceding an earthquake and decayed away exponentially following an earthquake, at rates that are determined by two separate parameters (see Supplementary Materials Figure S2 for a schematic representation of the model).

Each model was fitted by maximum likelihood in the R statistical environment (R Core Team, 2014, R Foundation for Statistical Computing, Vienna, Austria), using the nlminb function (which was found to converge more reliably than the optim function in this case). Optimisation was repeated 20 times using a range of starting values between −5 and 5 (drawn randomly from a uniform distribution). In 70% of cases, convergence was to a negative log-likelihood of 536.97, in 30% of cases a local optimum was found at 538.28, so the former was taken as the maximum likelihood. For the null model, the starting values were taken as the fitted values from the full model.

We then calculated Akaike’s Information Criterion (AIC) for each model, and compared the values to assess the evidence for an effect of earthquakes on large stranding events. AIC provides a way of selecting between different models by estimating the relative quality of each model constructed [24,25], where a lower AIC signifies the best model in terms of a reduction in information lost. We then constructed a 95% confidence interval (C.I.) for the parameter E using the profile likelihood technique, to determine an upper and lower plausible limit on how strong an effect of earthquakes might be at its peak. We then converted the estimate of E, and the endpoints of the 95% C.I. to an estimate of the proportion of large stranding events that were caused by earthquakes, as a more intuitive measure of the importance of earthquakes as a cause of large stranding events.

## 3. Results

### 3.1. Main Dataset

During the study period there were 5122 stranded animals (Table 1), with the most commonly stranded animals being Harbor seals *Phoca vitulina* and Californian Sea Lions, *Zalophus californianus*, which together made up more than half of all stranding events (Table 1).

During the period analysed (2005–2010), large stranding events (>5 marine mammals stranded) were reported on 235 days (10.2%), with an average of 8.2 animals stranding during such events (SD = 3.4, range = 6–38). There was strong evidence that the probability of a large stranding being reported on a given day differed among years (ΔAIC=128.6) with a systematic increase in reports over time (see Supplementary Materials Figure S1). There was also strong evidence of a difference among seasons (ΔAIC=214.8), with most large stranding events being reported in summer (June–August), then autumn (September–November), with very few being reported in winter (December–February) or spring (March–May) (see Supplementary Materials Figure S4). There was also evidence that a large stranding was more likely to occur if a large stranding had occurred on the previous day (ΔAIC=5.5; odds ratio = 1.60; 95% C.I. = [1.13, 2.27]). These findings support the inclusion of these variables in the model used to test for an effect of earthquakes on large stranding events.

**Figure 4:**
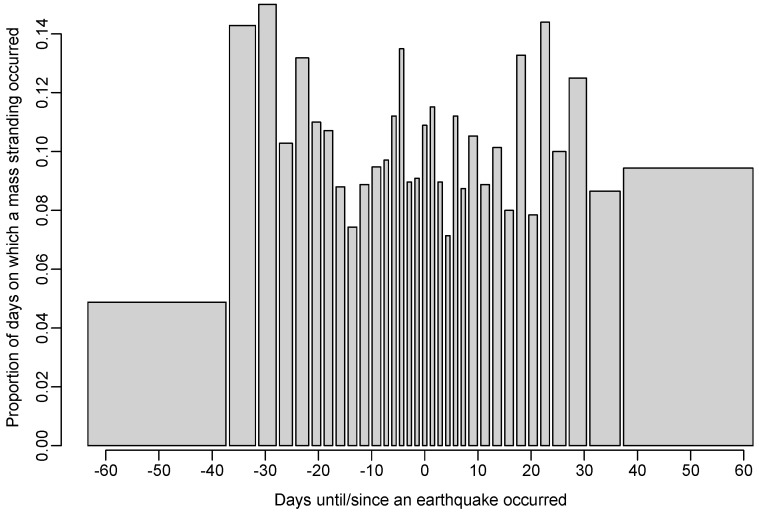
Bar plot of the proportion of days on which a mass stranding was recorded as a function of the number of days until the next earthquake (negative numbers) and the number of days since the previous earthquake (positive numbers). Categories are chosen such that there are at least 100 days in each.

**Figure 5:**
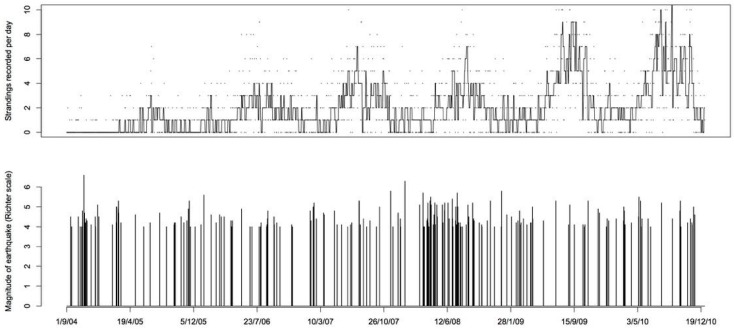
The top panel shows the time series of the number of standing events recorded each day, with a Tukey’s running median smoother. The bottom panel shows the earthquakes occurring on each day and their magnitude on the Richter scale.

The model (a) in which large stranding events were more likely to occur in temporal proximity to earthquakes was not supported relative to the null model (AIC increased by 1.3; see Table 2), indicating that the data provide little evidence for such an effect (see Figure 4 and Figure 5). Furthermore, our analysis suggests it is very unlikely that earthquakes are a major cause of marine mammal mass stranding events, with an estimated 0.35% of mass stranding events (95% C.I. = [0, 0.56]) being caused by earthquakes.

Further analyses (models b and c—see Supplementary Material Sections (b) and (c)) found little evidence of an effect of earthquakes on the probability of a stranding (of any number of animals > 0) occurring, or on the number of marine mammals stranding per day (see Table 2). In addition, 95% C.I.s showed that such effects, if they did exist, are likely very small and unimportant (see Table 2). When models were re-run including data from cetaceans only, there was still little evidence for an effect of earthquakes, and again the 95% C.I.s showed that such effects, if they did exist, are very small (see Table S1 in Supplementary Materials). Finally, a model (d) in which earthquakes decreased the probability a mass stranding would occur was also not favoured relative to the null model (AIC increased by 5.86).

### 3.2. Data Subset

**Figure 6:**
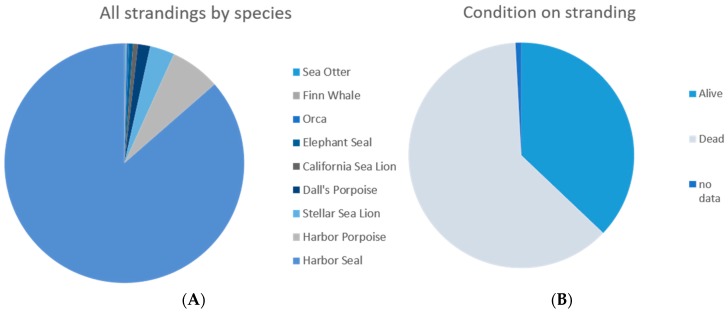
(**A**) Species breakdown, *n* = 604; (**B**) Stranding condition, *n* = 604.

Of 604 stranded animals in the data subset, the large majority were Harbor Seals (Figure 6). Of the total strandings, 224 stranded alive (37%) (Figure 6). Most (94.5%) of the live strandings were pups/calves with the remainder being mainly juveniles and prematures.

**Figure 7:**
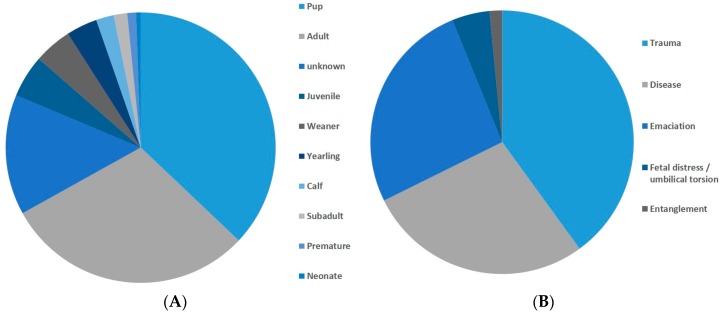
Dead stranded animals. (**A**) age classes. *n* = 375; (**B**) necropsy results *n* = 65.

Of the dead stranded animals, there was a mixture of age classes, but again, pups and juveniles were highly represented (Figure 7). Of those dead animals where a necropsy was performed and a possible cause of death stated, almost all died from disease, trauma, or were emaciated (Figure 7).

## 4. Discussion

Offshore earthquakes are, in the opinion of the authors, a plausible potential cause of some stranding events but this possible causative factor had not previously been systematically investigated. The current analysis attempted to empirically test the contribution of offshore earthquakes to stranding risk using a large dataset collected over six years. Although earthquakes are a source of sound and disturbance under the sea, and could cause stranding in various ways, such as disorienting the animals [12], frightening the animals into random directionless movement, damaging navigational capabilities, or by pre seismic cues warning animals to leave coastal areas [14], the analysis that we performed indicated that offshore earthquakes occurring in the Cascadia subduction zone did not influence the probability of single or large stranding events off the Washington and Oregon coasts.

The Marine Mammal Center [26] cites numerous reasons for marine mammal stranding, which can be roughly categorised by their type and origin and relate to:

1. anthropogenic causes such as shooting, entanglement, ingestion of debris, and collision with boats [2], as well as anthropogenic sound and naval activities [7];
2. predation by the animals’ natural predators (such as shark attacks);
3. disease, of which there are numerous varieties known to affect marine mammals such as herpes virus, Leptospirosis and others;
4. toxins (whether natural toxins or anthropogenic pollutants);
5. lack of food and/or inexperience of young animals; and, finally,
6. oceanographic or environmental causes, such as changes in prey distribution due to El Niño events.

Earthquakes fall into the latter category but they are not generally accepted as a significant cause of stranding, and our analysis supports this view. Given the large number of factors influencing marine mammal stranding, it could be predicted that earthquakes would be responsible for only a proportion of strandings, nevertheless, our study showed that the null model was always supported over a model, including earthquakes as a factor. Our primary analysis comprised a large dataset, of which many of the strandings probably had anthropogenic or other causes unrelated to earthquakes. Our analysis was robust enough that any influence of earthquakes would be revealed as an increase in stranding probability regardless of the presence of non-earthquake related stranding. We ran simulations where anthropogenic causes were assigned to 0%, 10% … … 90% of strandings to test the effect of this on the overall results of the model, and it was found that no matter how many strandings were caused by human factors, earthquakes still were not an important factor influencing stranding probability (for full details, see Supplementary Material, Section e). Furthermore, analysis of the data subset for which detailed information was available, showed that the overwhelming majority of live stranding animals were pups, juveniles, or premature (see cause 5, above) and that almost all dead stranded animals, where a cause was ascertained, were diseased, emaciated, or suffered from trauma, such as gunshot or boat strike (causes 1, 3, and 5), which would not be compatible with a cause of offshore seismicity.

Several studies have investigated stranding in the same or nearby geographical area to that used in our study. A 10-year study of California sea lions off the California coast, due South of the area we studied found that malnutrition was the most frequent cause of stranding events (32%), then leptospirosis (27%), trauma (18%), domoic acid intoxication (9%), and cancer (3%) [5], which supports the results that we obtained. El Niño events in certain years (1992, 1993, and 1998) led to more stranding events that were caused by malnutrition, while in other years, leptospirosis was a more important factor [5]. A study covering the Oregon coast (the area included in our study) over an earlier time period, performed an autopsy on numerous marine mammals to ascertain the cause of death and found that gunshot, trauma, and disease explained almost all of the stranding events [27], and again, this mirrors the results that we obtained.

In our study, sampling effort increased across years, which is likely to be due to an increased public awareness and government initiatives in formally reporting stranding events [27]. If there were any real association between earthquakes and mass stranding events then we would expect the pattern of mass stranding events within each year to follow the pattern of earthquakes. However, there was in fact a strong seasonal effect, with most stranding events being reported between June and November (Supplementary Materials Figure S4), which supports an analysis of stranding events along the same stretch of coastline between 1930 and 2002 [28]. The probable reasons for this seasonal effect relate to species’ seasonal movements, human activity, and oceanographic factors [28].

The study had several limitations, including only targeting one particular area of coastline for a limited period of time. No very large earthquakes (M > 6.5) occurred during the study period. It is possible that large earthquakes (M > 6.5) may cause marine mammals in the immediate vicinity to strand. Earthquakes were of various depths and distances from the coast, and this was not taken into account. We tested for temporal, not spatial correlation to earthquakes. However, the presence of more than 100 earthquakes and more than 4000 stranding events means that we are confident in accepting the null hypothesis. This is not to say that offshore earthquakes never cause marine mammals to strand, but for the study period and location, earthquakes were not found to be a significant risk factor for either single or large stranding events. As we looked both forward and backward in time from the dates of earthquakes, we have ruled out animals stranding due to some sort of precursory event occurring in the earthquake preparation zone, such as magnetic field shifts, gases released from the fault, positive ions, or changes in water chemistry that are reported to affect animals [14,29], as well as ruling out effects occurring from the earthquake itself.

## 5. Conclusions

Our study adds to the body of literature examining stranding in the context of environmental changes and natural hazards. Our findings are also important in addressing the many speculative accounts which purport, without evidence or based only on single observations, that earthquakes influence marine mammal stranding. Investigators should closely examine any reports of marine mammals stranding events that have been attributed to offshore earthquakes. It is certainly clear that there are a multitude of factors influencing the stranding of marine mammals and a single cause is unlikely to be responsible. Given the variety and complexity of factors influencing stranding events, earthquakes cannot convincingly be viewed as making an important or significant contribution to stranding risk, influencing just 0.35% of large stranding events (95% C.I. = [0, 0.56]). The authors reiterate the comments of Bradshaw et al. [19] in calling for rigorous hypothesis testing to be carried out, rather than speculation, to understand the causes of marine mammal stranding events.

## Figures and Tables

**Table 1:** The species of marine mammal stranded on the Washington and Oregon coasts from 2005 to 2010.

Order	Scientific Name	Common Name	Number Stranded
Carnivora	*Arctocephalus townsendi*	Guadalupe fur seal	41
	*Callorhinus ursinus*	Northern fur seal	36
	*Enhydra lutris*	Sea otter	2
	*Eumetopias jubatus*	Steller sea lion	411
	*Mirounga angustirostris*	Northern elephant seal	95
	*Phoca vitulina*	Harbor seal	2664
	*Zalophus californianus*	California sea lion	1186
Cetacea	*Balaenoptera acutorostrata*	Minke whale	2
	*Balaenoptera edini*	Bryde’s whale	2
	*Balaenoptera physalus*	Fin whale	5
	*Berardius bairdii*	Giant beaked whale	1
	*Delphinus delphis*	Short-beaked common dolphin	5
	*Eschrichtius robustus*	Gray whale	47
	*Globicephala macrorhynchus*	Short-finned pilot whale	1
	*Kogia* spp.	Various Sperm whales	2
	*Lagenorhynchus obliquidens*	Pacific white-sided dolphin	4
	*Lissodelphis borealis*	Northern right whale dolphin	2
	*Megaptera novaeangliae*	Humpback whale	13
	*Mesoplodon carlhubbsi*	Hubb’s beaked whale	1
	*Orcinus orca*	Killer whale	4
	*Phocoena phocoena*	Harbour porpoise	285
	*Phocoenoides dalli*	Dall’s porpoise	22
	*Physeter macrocephalus*	Sperm whale	4
	*Stenella coeruleoalba*	Striped dolphin	6
	*Tursiops truncatus*	Common bottlenose dolphin	2
	*Ziphius cavirostris*	Cuvier’s beaked whale	6
Unknown			273
Total			5122

**Table 2:** Statistical outcomes for single and large stranding events.

Response Variable	AIC (Akaike Weight)			Estimated % of Strandings/Mass Strandings Caused by Earthquakes [95% C.I.]
	No Effect	Positive Effect	Inhibitory Effect	
(a) Large stranding (binary)	1100.69	1101.94	1106.55 (3%)	0.35% [0, 0.56]
	(63%)	(34%)		
(b) Stranding (binary)	2178.86	2181.66	NA	1.77% [0, 3.43]
	(80%)	(20%)		
(c) Stranding (count)	8552.06	8558.06	NA	0.00% [0, 6.00]
	(95%)	(5%)		

## Supplementary Materials

The following are available online at http://www.mdpi.com/2076-2615/8/2/18/s1, Figure S1: Number of stranding events (top panel) and large stranding events (bottom panel) recorded by year, Figure S2: Schematic representation of the statistical model used. The probability of a large stranding is assumed to be at its peak on the day of the earthquake, building up exponentially in the preceding period and decaying away exponentially following the earthquake. In this hypothetical example, the effect builds up faster than it decays away, but this is not constrained to be the case, Figure S3: Schematic representation of the statistical model assuming an inhibitory effect of earthquakes on large stranding events. The inhibition of large stranding events is assumed to be at its peak on the day of the earthquake, building up exponentially in the preceding period and decaying away exponentially following the earthquake. In this hypothetical example, the effect builds up faster than it decays away, but this is not constrained to be the case, Figure S4: Proportion of days on which at least one large stranding was recorded, by season. Error bars show 95% confidence intervals, Figure S5: Proportion of simulations in which the 95% confidence intervals (C.I.s) contained the true value of E (= −3) as a function of the proportion of strandings excluded as having other (non-earthquake) causes. The dashed line shows 0.95, the ideal for perfect performance of the 95% C.I.s. Error bars show Wilson’s 95% confidence intervals for the plotted proportions, Table S1: Reanalysis using only data on cetaceans.
