# Supplementary material for: Offshore Earthquakes Do Not Influence Marine Mammal Stranding Risk on the Washington and Oregon Coasts
Source: Animals (Basel). 2018 Jan 26;8(2):18. doi: 10.3390/ani8020018 (PMC5836026; doi:10.3390/ani8020018)
Supplement: Supplementary file 1 [file animals-08-00018-s001.pdf]

Supplementary Material

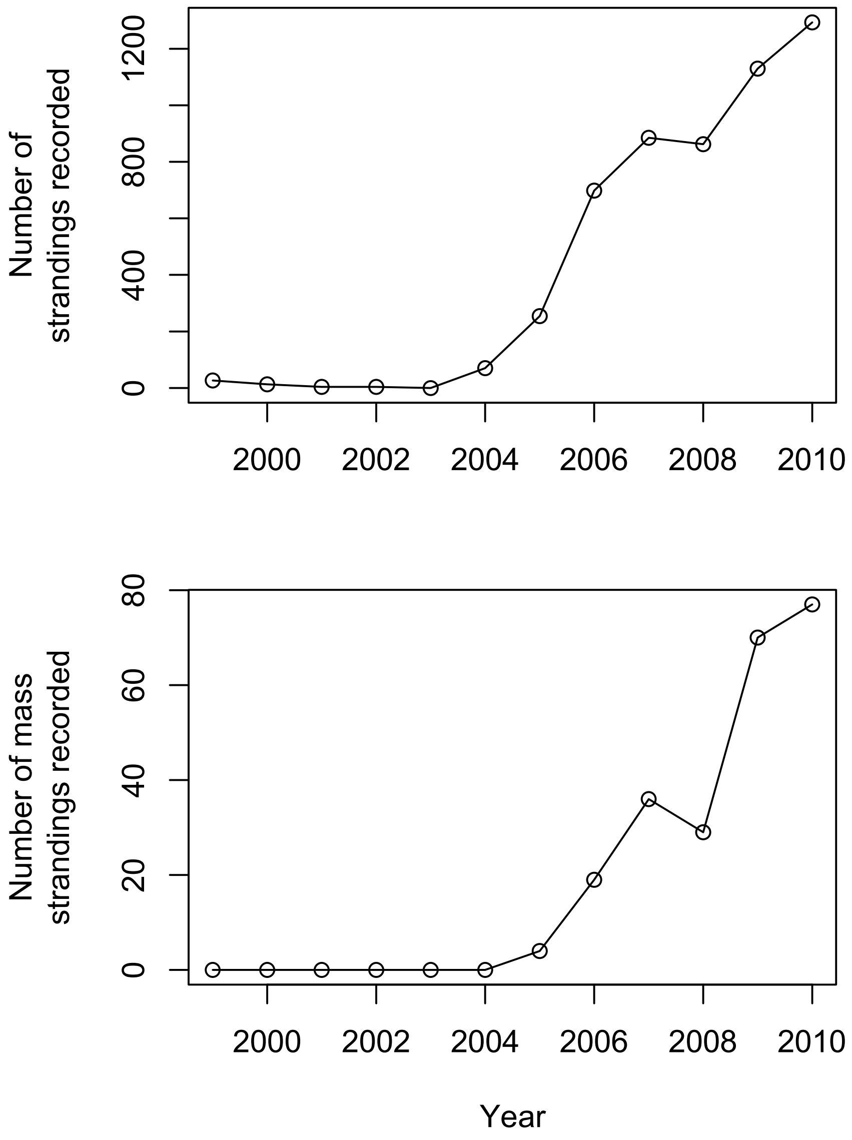

Figure S1. Number of stranding events (top panel) and large stranding events (bottom panel) recorded by year.

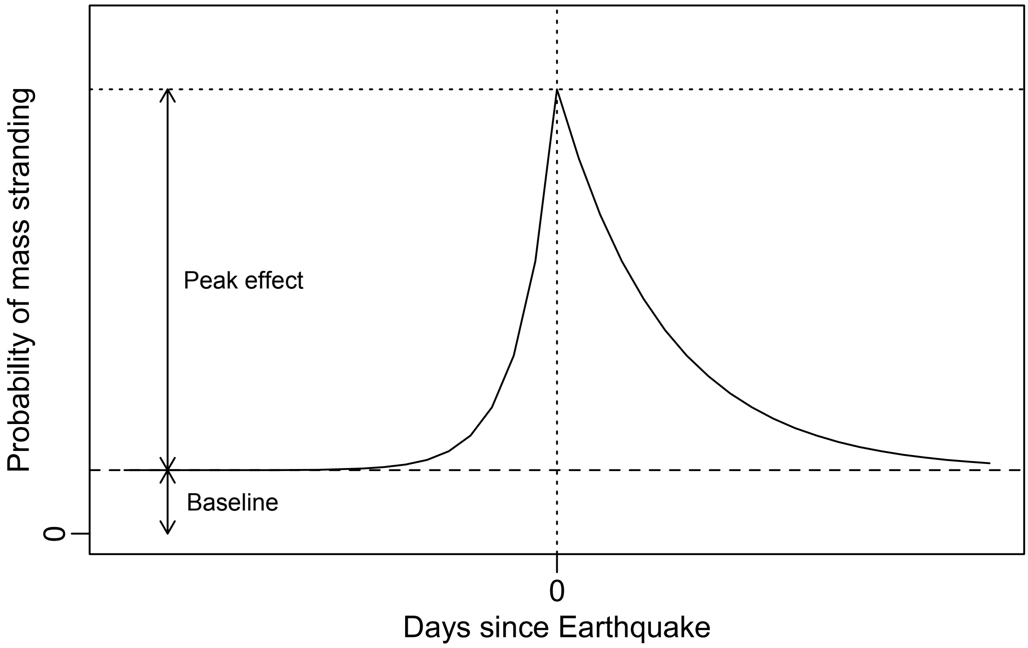

Figure S2. Schematic representation of the statistical model used. The probability of a large stranding is assumed to be at its peak on the day of the earthquake, building up exponentially in the preceding period and decaying away exponentially following the earthquake. In this hypothetical example, the effect builds up faster than it decays away, but this is not constrained to be the case.

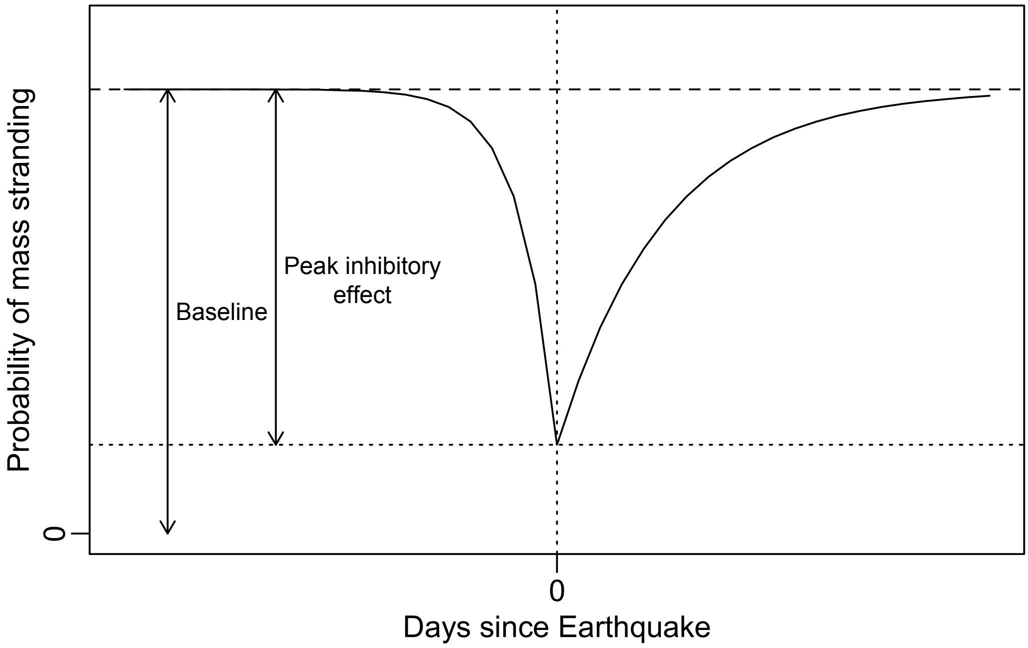

Figure S3. Schematic representation of the statistical model assuming an inhibitory effect of earthquakes on large stranding events. The inhibition of large stranding events is assumed to be at its peak on the day

of the earthquake, building up exponentially in the preceding period and decaying away exponentially following the earthquake. In this hypothetical example, the effect builds up faster than it decays away, but this is not constrained to be the case.

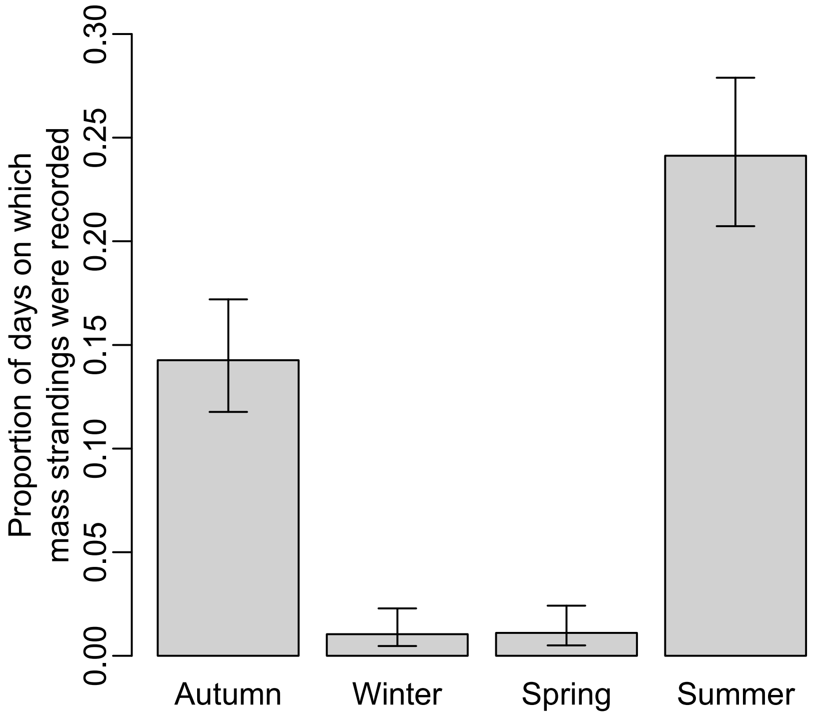

Figure S4. Proportion of days on which at least one large stranding was recorded, by season. Error bars show 95% confidence intervals.

Model description

We fitted models to three dependent variables: a) whether at least one large stranding was reported on each day (binary- see also model d) below); b) whether at least one stranding was reported on each day (binary); and c) the number of mammals reported stranded on each day (count). All models had a similar form, so here we first describe the model for a) before describing how this was modified for b) and c).

a) Model of large stranding binary variable

We take $M_n = 1$ when there was one or more large stranding events on day n in year i and season j, and $M_n = 0$ otherwise. We assumed that there was a baseline probability, b_{nij}, a large stranding would occur on day n in year i in season j, in the absence of any effect of earthquakes, which was determined using the standard logistic model used in a generalized linear model:

$$b_{nij} = \frac{\exp(Y)}{1+\exp(Y)}$$

where,

$$Y = \alpha_i + \beta_j + \gamma M_{n-1}$$

Here α_i allows for differences in stranding between years; β_j allows for seasonal differences, where 1 = autumn, 2 = winter, 3 = spring and 4 = summer, and $\beta_1 = 0$ to avoid overparameterisation; γ is a parameter allowing for the possibility that if there is a large stranding on one day, there is more likely to be one the following day, i.e. autocorrelation.

We then assume that on top of the baseline probability a large stranding will occur, there is also a probability of one or more large stranding events caused by seismic activity associated with earthquakes, e_{nij}:

$$e_{nij} = \frac{\exp(E)}{1+\exp(E)} max\left(exp(-\delta t_{b,n}), exp(-\varepsilon t_{a,n})\right)$$

Where $t_{a,n}$ is the days since the most recent previous earthquake, and $t_{b,n}$ is the days until the next earthquake, and δ and ε are positive parameters fitted to the data. If there is an earthquake on day n then $t_{b,n} = t_{a,n} = 0$, meaning the expression reduces to exp(E)/(1+exp(E)). Thus the parameter E is the log odds of one or more large stranding being caused by an earthquake on the day the earthquake occurs. The model assumes that this effect exponentially decays away after an earthquake has occurred at a rate determined by ε, and likewise that the effect builds up prior to an earthquake occurring, at a rate determined by δ. If day n is sandwiched between two earthquakes, the effect is set for whichever earthquake exerts the strongest effect, i.e. the maximum of $exp(-\delta t_{b,n})$ and $exp(-\varepsilon t_{a,n})$.

Since the event $M_n = 1$ denotes that at least one large stranding has occurred on day n, the probability the event occurs is given by:

$$p(M_n = 1) = b_{nij} + (1 - b_{nij})e_{nij}$$

The log-likelihood for the data is given as:

$$\log(L) = \sum_n log\left(b_{nij} + (1 - b_{nij})e_{nij}\right)$$

The model was fitted by maximum likelihood in the R statistical environment, using the optim function. We then calculated Akaike's Information Criterion (AIC) for the model, and compared this to the AIC for model in which there was effect of earthquakes, i.e. $e_{nij} = 0$ for all n, in order to assess the evidence for an

effect of earthquakes on large stranding events. We then constructed a 95% confidence interval for E using the profile likelihood technique to determine an upper and lower plausible limit on how strong such an effect might be at its peak.

We then converted the maximum likelihood estimator (MLE) for E to an estimate of the proportion of large stranding events that were caused by earthquakes. We did this by calculating the probability that each observed large stranding was caused by an earthquake, given by:

$$\frac{e_{nij}}{b_{nij} + (1 - b_{nij})e_{nij}}$$

and substituting the MLEs into the model. Summing these across all large stranding events gives the estimated number for the number of large stranding events caused by earthquakes, and dividing by the total number of large stranding events gives the required proportion. We then repeated the calculation twice, replacing the MLE for E with for the endpoints of the 95% confidence interval for E, thus obtaining a plausible range of values for the proportion of large stranding events caused by earthquakes.

b) Model of stranding events binary variable

We repeated the analysis given in a) above, but replaced M_n with S_n, where $S_n = 1$ if there was at least one stranding reported on day n and $S_n = 0$ otherwise.

c) Model of number of stranding events per day

The model had a similar form to that given in a) above, but we replaced M_n with C_n, the number of stranding events reported on day n. Here

$$C_n \sim Pois(\mu_{nij})$$

where

$$\mu_{nij} = b_{nij} + e_{nij}$$

Now b_{nij} gives the expected number of stranding events on day n not caused by earthquakes, determined using a log link function as in a standard Poisson GLM:

$$b_{nij} = \exp(Y)$$

and e_{nij} gives the expected number of stranding events on day n caused by earthquakes, determined in an analogous fashion as for a) above:

$$e_{nij} = \exp(E) max\left(exp(-\delta t_{b,n}), exp(-\varepsilon t_{a,n})\right)$$

Thus *exp(E)* now gives the expected number of stranding events caused by an earthquake on the day the earthquake occurs, with ε and δ again determining how quickly the effect decays and builds up respectively.

Model fitting, parameter estimation and model comparison proceeded as for model a), and the proportion of stranding events caused by earthquakes was estimated from the fitted model in a manner analogous to that used for model a) above.

d) Potential inhibitory effect of earthquakes on large stranding events

We modified model a) to test for a potential inhibitory effect of earthquakes on large stranding events, by setting:

$$p(M_n = 1) = b_{nij}(1 - e_{nij})$$

Thus exp(E)/(1+exp(E)) now gives the proportional reduction in the probability at least one large stranding will occur on the day an earthquake occurs. e.g. if E=0, then exp(E)/(1+exp(E)) = 0.5, meaning large stranding events are half as likely on the day of an earthquake occurs that if the earthquake had not occurred. ε and δ again determine how quickly the effect decays and builds up respectively.

e) Interpretation of non-significant results in the model

With any statistical model or test, a non-significant effect does not constitute evidence that an effect does not exist in reality. So the fact that we find no significant effect of earthquakes does not mean that

earthquakes have no effect on marine mammal strandings. Nonetheless, we are able to conclude that the effect is, if present at all, a small and unimportant effect. We do this by using the model and data to provide an upper plausible limit on the effect earthquakes have, by constructing 95% confidence intervals. If a) the upper limit of the 95% confidence interval is small, and b) the 95% confidence intervals are valid or approximately valid, we have good evidence that the effect of earthquakes on marine mammals is, if present at all, a small and unimportant effect. In the main text we show that a) is true. Here we assess the validity of the 95% confidence intervals. 95% confidence intervals are valid if they contain the true value 95% of the time, and approximately valid if they contain the true value close to 95% of the time.

The profile likelihood technique for constructing confidence intervals is a standard and accepted technique in statistical modelling [29]. However, concerns might be raised as to whether the statistical model presented here validly estimates the effect of earthquakes, if one does not exclude cases that are known to be caused by other factors, as was the case in this study. Here we present simulations showing that the validity of the 95% confidence intervals is not affected by exclusion of strandings from the data as being the result of other causes, even when no strandings are excluded from the study.

We simulated data from the model presented above, such that there were an equal number of strandings as in the real data. Parameter values were taken from those estimated from the real data, except for those determining the effect of earthquakes, since this effect was found to be negligible in the models fitted to the real life data. Therefore, we used a larger effect in the simulations (E = -3; $\delta = \varepsilon = 0.1$), to investigate whether a real and important effect could be reliably estimated without exclusion of strandings caused by other cases. Following the model, we assigned each stranding as caused by an earthquake with probability $e_{nij}/(e_{nij} + b_{nij})$. We then analysed the resulting data set excluding {0%, 10%,… …90%) of strandings that were not caused by earthquakes, representing exclusion of strandings with known causes under differing levels of knowledge about the other (non-earthquake) causes of strandings. We then recorded whether the 95% confidence interval for E contained the true value (E = -3) in each case. We then repeated the simulation 1000 times to calculate the proportion of times that the true value of E was within the 95% confidence intervals (expected to be close to 0.95 if the procedure is valid).

We found that the proportion of cases in which the 95% confidence intervals contained the true value was not affected by the proportion of strandings excluded as having another known cause, even when no such cases were excluded (see Fig. S5). Performance is slightly below 95% (~93%) due to the approximate nature of profile likelihood confidence intervals (the same is true of alternatives e.g. Wald C.I.s). However, this is likely to be the case in many applications of this standard statistical technique. This shows that the 95% C.I.s derived for the real data are valid enough to consider them as providing an upper plausible limit on the effect being estimated. We thus conclude that we have good evidence that the effect of earthquakes on marine mammals is, if present at all, a small and unimportant effect.

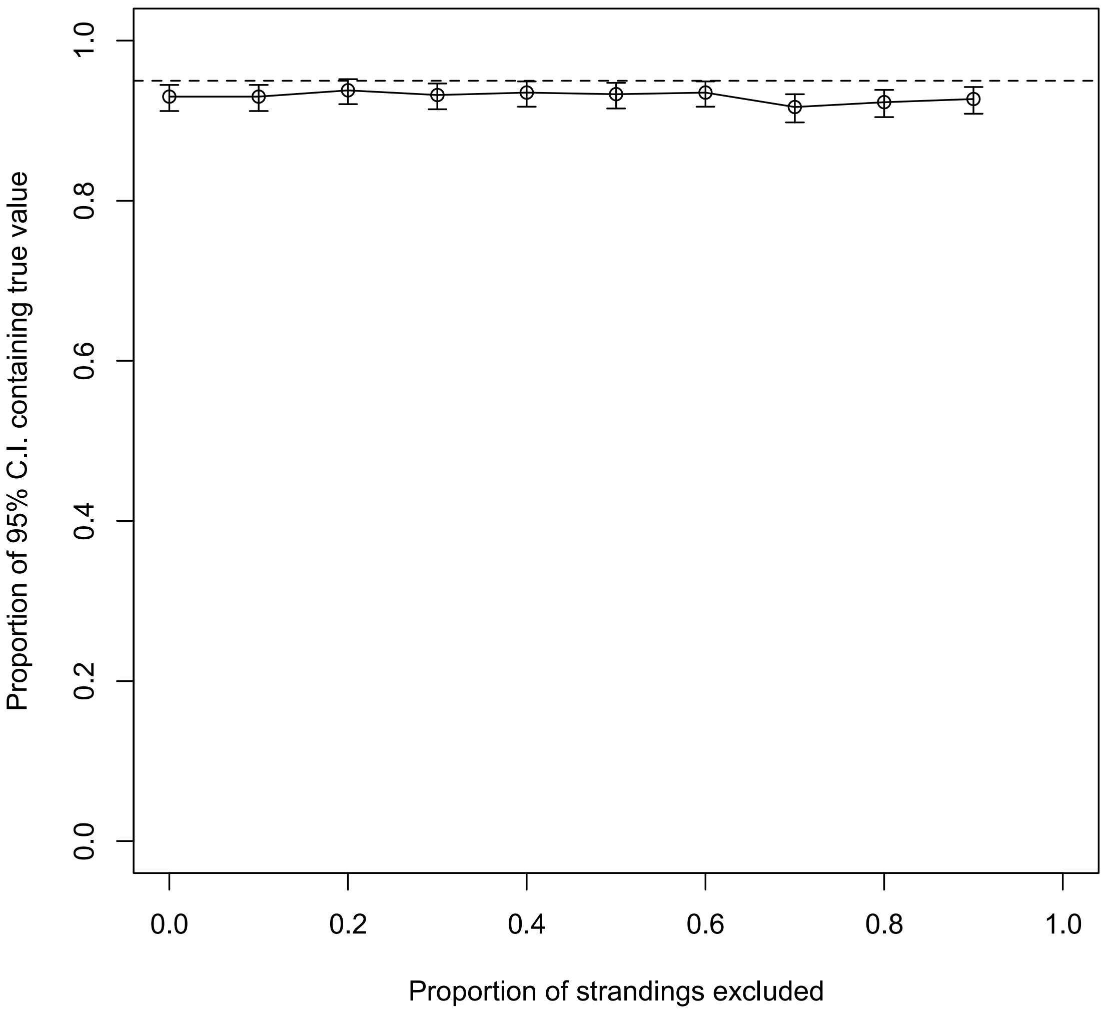

Figure S5. Proportion of simulations in which the 95% confidence intervals (C.I.s) contained the true value of E (= -3) as a function of the proportion of strandings excluded as having other (non-earthquake) causes. The dashed line shows 0.95, the ideal for perfect performance of the 95% C.I.s. Error bars show Wilson's 95% confidence intervals for the plotted proportions.

Table S1. Reanalysis using only data on cetaceans.

Response Variable	Estimated % of Strandings/Mass Strandings Caused by Earthquakes [95% C.I.]
(a) Large stranding (binary)	NA*
(b) Stranding (binary)	1.77% [0, 3.43]
(c) Stranding (count)	3.72% [0, 28.6]

*Very few strandings with >5 cetaceans meant this model could not be fitted.
